# TiO2 nanoparticles induced micronucleus formation in human liver (HepG2) cells: comparison of conventional and flow cytometry based methods

**DOI:** 10.1186/1755-8166-7-S1-P79

**Published:** 2014-01-21

**Authors:** NV Srikanth Vallabani, Ritesh K Shukla, Dinesh Konka, Ashutosh Kumar, Sanjay Singh, Alok Dhawan

**Affiliations:** 1 Institute of Life Sciences, School of Science and Technology, Ahmedabad University, Ahmedabad –Gujarat, India

**Keywords:** Titania, Micronucleus, Nanotoxicity, Flow cytometry

## Background

TiO_2_ nanoparticles (TiO_2_ NPs) are extensively used metal oxide NPs in cosmetics, as an additive in pharmaceuticals, food colorant etc. Being widely used NPs, their toxicity assessment studies help in understanding the adverse effects to the humans. It is likely that NPs exposure to the humans can be through different routes but will finally reach the liver. Therefore, an attempt was made to explore the genotoxicity of TiO_2_ NPs on human liver cells (HepG2).

## Materials and methods

TiO_2_ NPs were characterized by transmission electron microscopy (TEM) for their primary size, shape and dynamic light scattering for their size, size distribution and zeta potential in culture medium. Cellular uptake of TiO_2_ NPs in HepG2 cells was detected using flow cytometry method. Moreover, ultrathin sections of cells were analysed using TEM to visualise the internalisation of TiO_2_ NPs. The genotoxic potential of TiO_2_ NPs was assessed by micronucleus assay using the conventional and flow cytometry methods.

## Results

TEM analysis of TiO_2_ NPs revealed a mean diameter size of 30-70 nm and DLS measurements showed a mean hydrodynamic diameter and zeta potential of 192.5 ± 10 nm and -11.4 ± 1.2 mV, respectively. The electron microscopy and flow cytometry studies for particle internalisation showed a significant cellular uptake of TiO_2_ NPs in the human liver cells (HepG2). A significant (p < 0.05) induction in micronucleus formation was observed at 20 µg/ml of TiO_2_ NPs exposure when compared to control cells. However further treatment to HepG2 with higher concentrations (40 and 80 µg/ml) showed a decrease in micronucleus formation than 20 µg/ml.

In contrary, the flow cytometric results exhibited a significant concentration dependent induction of micronucleus in HepG2 cells exposed to all concentrations (20, 40 and 80 µg/ml) of TiO_2_ NPs than control.

## Conclusion

The difference in the micronucleus frequency obtained from conventional and flow cytometry methods in HepG2 may be due to the accumulation of TiO_2_ NPs on prepared slides, which hinders the counting of micronucleus (in conventional method). However, in the flow cytometry analysis, these nanoparticles do not interfere with the optics. Hence, it is proposed that in the case of NPs treatment, flow cytometry based micronucleus assay should be used instead of the conventional method.

